# New York State Inebriate Asylum

**Published:** 1858-11

**Authors:** 


NEW YORK STATE INEBRIATE ASYLUM.—The corner-stone of this institution was laid on the 24th of September with ceremonies and addresses from several distinguished gentlemen, including Rev. Dr. Bellows, Dr. John Francis, and Hon. Edward Everett. So far as we know this is the first instance of anything of the kind having been undertaken in this country, and we are glad to say that it is due in great part to the efforts and enterprise of the medical profession. Its object is to reclaim confirmed drunkards, to keep them from temptation, and rid society of their presence. It is not expected that the institution will be opened for two years; in the mean while we wish the directors the best success in the accomplishment of their noble undertaking.

